# Correlation Clustering of Stable Angina Clinical Care Patterns for 506 Thousand Patients

**DOI:** 10.1155/2017/6937194

**Published:** 2017-11-14

**Authors:** Zsolt Vassy, István Kósa, István Vassányi

**Affiliations:** ^1^Medical Informatics Research and Development Centre, University of Pannonia, Veszprém, Egyetem u. 10 8200, Hungary; ^2^Department of Medical Rehabilitation and Physical Medicine, University of Szeged, Szeged, Korányi fasor 8-10 6720, Hungary

## Abstract

**Objectives:**

Our goal was to apply statistical and network science techniques to depict how the clinical pathways of patients can be used to characterize the practices of care providers.

**Methods:**

We included the data of 506,087 patients who underwent procedures related to ischemic heart disease. Patients were assigned to one of the 136 primary health-care centers using a voting scheme based on their residence. The clinical pathways were classified, and the spectrum of the pathway types was computed for each center, then a network was built with the centers as nodes and spectrum correlations as edge weights. Then Louvain clustering was used to group centers with similar pathway spectra.

**Results:**

We identified 3 clusters with rather distinct characteristics that occupy quite compact spatial areas, though no geographical information was used in clustering. Network analysis and hierarchical clustering show the dominance of medical university clinics in each cluster.

**Conclusion:**

Though clinical guidelines provide a uniform regulation for medical decisions, doctors have great freedom in daily clinical practice. This freedom leads to regional preferences of certain clinical pathways, the intercenter professional links, and geographical locality and coupled with quantifiable consequences in terms of care costs and periprocedural risk of patients.

## 1. Introduction

Publicly financed health care is a special segment of the economy, in which the utility and the cost of individual procedures frequently diverge. Patients are maximally interested in the most effective services and are blind for the expenses, while physicians have a similar preference for effectiveness, but a heterogeneous sensitivity for the expenses of applied services. In well-controlled health-care systems, this latter heterogeneity can be minimized, but in Hungary, the country investigated in this paper, the control is dominantly of administrative type, so physicians have a relatively great freedom regarding the chosen treatment. The clinical practice is regulated by scientific guidelines, but the limited effect of such guidelines on the clinical practice is well documented [1, 2].

On the other hand, the systematic recording of performed procedures in the publicly financed healthcare generated rapidly growing electronic biomedical databases. If suitable, innovative data mining and analysis methods are employed to leverage this data; the resource allocation and the overall quality of healthcare delivery can be improved. Our working group already evaluated earlier the characteristics of patients referred to the first investigation in different areas of the country [3] and documented the systematic bias due to factors like geographical distance to the invasive diagnostic centers [4, 5] or local volume capacity of invasive diagnostics [6].

It is not so easy, however, to depict the complex pattern of patient evaluation pathways consisting of a time series of investigations and procedures. Interactive tools and visualization have been proposed for mining clinical event patterns in [7]. Another possible approach is the network-based representation and analysis of data, a widely used method in the social and business sciences to both visualize and identify the components as well as their structure and interactions [8–10], in some cases applied also in the health domain [11], but not yet for the study of the interactions among clinical care providers.

Network-based analysis often relies on clustering, a method of grouping a set of objects in such a way that objects in the same group or cluster are more similar (in one or more characteristics) to each other than to those in other clusters. Clustering, a standard method of business intelligence, has already been successfully and innovatively applied to the biomedical data, cases, trials, clinical models, and other entities of the health-care domain [12–16]. In this paper, we present our results using a network science-based approach in the field of health-care pathway analysis.

## 2. Methods

The proposed method can be briefly outlined as a sequence of the following steps:

1. Data cleaning and classification of care events
2. Assigning a dominant “de facto” care provider to each ZIP area by a voting scheme
3. Forming event series using the events of the same patient and classifying the series in one of the 15 distinct series types
4. Computing the event series spectrum for each provider and the correlation among providers based on the series spectra
5. Building a network of the providers using the correlations as edge definition
6. Cluster and analyze the network using standard network science methods

The steps are detailed below.

### 2.1. Data Preparation and Cleaning

The basic source of the data was the Hungarian national health-care reimbursement register run by the National Healthcare Services Center (ÁEEK) from which we queried the patients who underwent ischemic heart disease- (IHD-) related diagnostic procedures between 1 January 2004 and 31 December 2008 in outpatient or inpatient care, a total of 506,087 patients. The case data contained the recorded diagnoses and procedures, excluding cases with acute myocardial infarction (AMI). We categorized the care events of a case based on the International Classification of Disease (ICD) codes and International Classification of Procedures in Medicine (ICPM) related to each event, and we also created an event from each death case. This resulted in a time-stamped event list for each patient. For a more detailed description of the categorization scheme, please see the Appendix of [3].

In the next phase, we merged some events in the event list according to a set of rules to eliminate redundant (phantom) events due to the known common practice of coding the relevant procedures (e.g., two-day single photon emission computed tomography protocol). Since we wanted to focus on patients with stable conditions at the time of onset, we considered only patients who had at least 180 days long event-free period followed by an “index” event. The rules applied for qualifying an event as index are detailed in the Supplement of [4] along with other details of the data cleaning process. For each such patient, we defined the “event series” as the part of the event list that started with the index event and ended by the next 180 days long event-free period, death, or the end of the observation period.

Since the basic objective of this work was the characterization of the professional behavior patterns of the care providers, we distinguished three different types of care procedures:

- “E” type: noninvasive, nonimaging investigations, that is, stress electrocardiography
- “NI” type: noninvasive imaging investigations like single photon emission computed tomography (SPECT) and stress echocardiography
- “I” type: invasive procedures like coronary angiography (CA), percutaneous coronary intervention (PCI), or coronary artery bypass grafting (CABG)

Invasive procedures require a special attention because they are generally more risky and more expensive than the noninvasive ones. The clinical pathways were then built up from a combination of events of these three types, all other events were excluded from the analysis. We considered E type events as belonging to the “primary” care, NI type events to the “secondary” care, and I type events to the “tertiary” care.

In the next step, we identified the dominant de facto primary care center for each ZIP area using the patients' residential ZIP code and a simple voting scheme based on the patients' first stress electrocardiography in the observation period, so each patient with at least one event had a single vote. In order to tackle the large number of providers that appear in the reimbursement database, we considered the various departments of a large institution (e.g., a municipal hospital) with the same entity. This process yielded 136 de facto primary care centers. The same procedure was repeated for NI- and I-type events to identify the secondary and tertiary care providers, respectively [4].

The formation of the event series as described above and the identified de facto care centers were our earlier results and formed the starting point of the work presented in this paper. Our new contribution consists of three parts:

1. Classification of the event series and the characterization of care centers
2. Building a network of care centers based on event series profile correlations
3. Cluster analysis of the network of centers

### 2.2. Characterization of Care Centers

We computed an event series type flag for each event series based on the relative order of the first “E,” “I,” and “NI” events starting from the index event. For example, NI-I type means an NI-type event followed by an I-type event, but not preceded by an E-type one in the event list. We considered all of the 15 possible event series types, that is, “E,” “E-NI,” “E-NI-I,” “E-I,” “E-I-NI,” “NI,” “NI-E,” “NI-E-I,” “NI-I,” “NI-I-E,” “I,” “I-E,” “I-E-NI,” “I-NI,” and “I-NI-E.”

According to the general practice and guidelines [17], the expected clinical pathway is E-NI-I, but the physicians have the freedom to skip the E or NI steps for patients with a higher coronary artery disease risk or due to inability to perform the noninvasive imaging or nonimaging tests.

Table 1 shows an overview of the distribution of the 15 event series types. In the vast majority of cases, patients had only a single cardiac stress test (E). One-year mortality is naturally increased for those event series that start with an invasive event.

Since the average cost of the treatment is also an important feature of the care system, we computed the estimated cost for each individual event series. The calculation was based on the official reimbursement costs of the diagnostic as well as therapeutic events that appeared in the event series. Since slight yearly variations in these costs appeared over the study period, we used averaged values.  Table 2 shows the costs of the six basic event types in national currency (HUF) as well as Euro, at the currency exchange rate of December 2008.

In the next step, we aggregated the number of the occurrences of the various event series types for each care center and used the relative ratios of the various types of event series to characterize the centers.

### 2.3. Network Building

The primary care centers were compared with each other using Pearson's correlation according to the distribution of different clinical pathways. Pearson's correlation coefficient for a dataset {*x*1,…, *xn*} containing *n* values and another dataset {*y*1,…, *yn*} containing *n* values was calculated according to the following formula:

(1) $$\begin{array}{c} r=\frac{n\;\sum_{i=1}^{n}x_{i}y_{i}-\sum_{i=1}^{n}x_{i}\sum_{i=1}^{n}y_{i}}{\sqrt{\left(n\sum_{i=1}^{n}x_{i}^{2}-{\left(\sum_{i=1}^{n}x_{i}\right)}^{2}\right)\left(n\sum_{i=1}^{n}y_{i}^{2}-{\left(\sum_{i=1}^{n}y_{i}\right)}^{2}\right)}}=\frac{\sum_{i=1}^{n}\left(x_{i}-\bar{x}\right)\left(y_{i}-\bar{y}\right)}{\sqrt{\sum_{i=1}^{n}{\left(x_{i}-\bar{x}\right)}^{2}\sum_{i=1}^{n}{\left(y_{i}-\bar{y}\right)}^{2}}}, \end{array}$$

where $\bar{x}=1/n\sum_{i=1}^{n}x_{i}$ is the sample mean. The same holds for $\bar{y}$. In our case, *n* = 15 as we have 15 relative occurrence rates for the 15 event series types in each center.

The correlation matrix of 136 clinical pathway distributions of health-care centers *X*_1_,…, *X*_136_ is the 136 × 136 matrix, whose *i*,*j* entry is corr(*X_i_*, *X_j_*) Pearson's correlation coefficient. The correlation matrix is symmetric because the correlation between *X_i_* and *X_j_* is the same as the correlation between *X_i_* and *X_j_*. We calculated all of the coefficients with a 95% confidence level.

We made a network based on this correlation matrix in which nodes are primary care centers and edge weights are linearly transformed correlation coefficients. The transform was necessary because the network contained negative edge weights. Since most clustering methods, such as modularity-based methods, cannot handle negative weights, we transformed the correlation matrix into the edge weight matrix using the following simple linear transform:

(2) $$\begin{array}{c} w_{ij}=c_{ij}+2, \end{array}$$

where *w*_*ij*_ represents the edge weight of the edge between *i* and *j* nodes (primary health-care centers), and *c*_*ij*_ denotes Pearson's correlation coefficient between *i* and *j* nodes. The constant 2 was applied in (2) to eliminate the 0 values.

We also tried several other linear and nonlinear transforms like  (*c*_*ij*_ + 2)^∗^100, (*c*_*ij*_ + 2)^2^, or (*c*_*ij*_ + 2)^3^ in order to amplify the differences between health-care centers, but in all cases, the resulting clusters were nearly the same.

### 2.4. Network Clustering

Since the number of nodes of the generated network was small but the network was extremely dense, a modularity-based algorithm, the Louvain method was chosen for network clustering [18, 19]. This method is a simple and is an efficient method for modeling communities, that is, clusters of closely connected nodes, in large networks. The method is a greedy optimization method that attempts to optimize the modularity of a partition of the network. Modularity functions were introduced by Newman and Girvan [20, 21]. The modularity is a scalar value between −1 and 1 that measures the density of links inside communities as compared to links between communities. The modularity function can be written as follows:

(3) $$\begin{array}{c} Q=\frac{1}{2m}\sum_{i,j}\left(A_{ij}-\frac{k_{i}k_{j}}{2m}\right)\delta \left(c_{i},c_{j}\right), \end{array}$$

where

- *c*_*i*_ denotes the community (cluster) which node *i* has been assigned
- *A*_*ij*_ represents the weight of edge between *i* and *j*; if there is no edge then *A*_*ij*_ = 0
- *k*_*i*_ is the sum of the weights of the edges attached to node *i*
- *δ*(*u*, *v*) function is 1 if *u* = *v* and 0 otherwise

(4) $$\begin{array}{c} m=\frac{1}{2}\sum_{i,j}A_{ij}. \end{array}$$

In order to generate the Louvain clusters, we used the modularity optimizer tool [22] with the default settings and the following parameters:

- Number of random starts: 10
- Number of iterations: 10

### 2.5. Hierarchical Clustering and Opinion Leaders

We used the same clustering method (i.e., Louvain clustering) with the same parameters on the subgraphs that formed the clusters of the first level clustering as a hierarchical clustering method to identify second level clusters. In a similar manner, we used again the same clustering method with the same parameters inside the second level clusters to identify the third level clusters.

Using classical social network analysis techniques [23, 24], we also analyzed the importance of nodes for the network to identify the “opinion leaders.” For this purpose, we calculated the “degree” and “betweenness centrality” network centrality measures [25] on the whole health-care center network and on the subnetworks of the first level clusters.

### 2.6. Revascularization Rate

In order to characterize the clusters, we also computed the revascularization rate, a feature that shows the invasive nature of the care methodology. Revascularization is the common name of the invasive PCI and CABG procedures, both of which are used to restore the perfusion. The revascularization rate is the ratio of those cases in which CA procedure was followed by revascularization within 180 days, compared to the total number of cases with CA. This rate can be used as an index for the rationale behind referring the patient for CA, a potentially life-threatening and costly examination. If this index is extremely low compared to the average, then an unreasonably high proportion of patients was referred to CA.

### 2.7. Data Processing, Statistical Analysis, and Data Visualization Tools

For data preparation and data cleaning, we used the Microsoft SQL Server 2012 database management system [26]. All statistical analyses were performed using the R 3.1.1 tool [27]. We used Fisher's exact test to determine statistical significance. A *p* value <0.05 was considered statistically significant for all analyses.

For mortality rate standardization, we used direct standardization [28]. Calculation of network centralities and network visualization was performed using Gephi 0.9.1 [29].

The spatial map was produced using the Quantum GIS 2.8 open source software package [30]. The Louvain clustering method and smart local moving algorithm were performed using the modularity optimizer tool [29]. The ModuLand network modularization method was run on our network with the ModuLand plug-in of Cytoscape 2.8.2 [31].

## 3. Results

We built the correlation matrix and the network of the 136 health-care centers based on Pearson's correlation coefficients. Using Louvain clustering in this network, 3 first level health-care center groups were identified.

**Figure 1:**
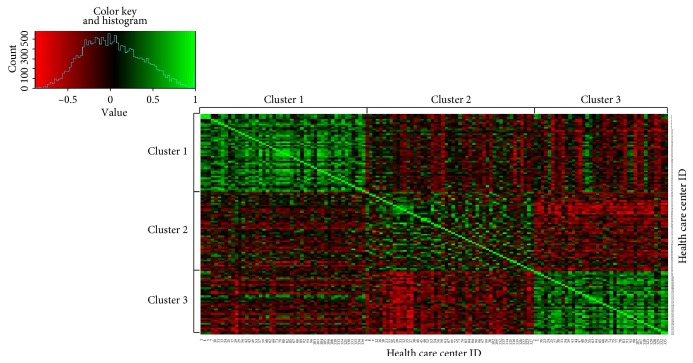
Heat map of the correlation matrix of health-care centers grouped in three first level clusters. See explanation in the text.

Figure 1 displays the “heat map” of correlations among health-care centers grouped by clusters. Each center has a corresponding row and column, and the colored patch at the intersection of a center's row with another's column represents the correlation between the two centers' pathway distribution. We used a color range from red over black to green, red representing negative, black neutral, and green positive correlation. The centers belonging to the same cluster are placed next to each other, so the figure shows the internal structure of the cluster as well as the intercluster relations. The color key shows the distribution of the correlation values over the whole matrix as a continuous white line.

It is clear from the figure that the strongest intracluster connections, that is, the strongest green patches, appear in cluster 1 and that cluster 2 is the most diffused (i.e., least characteristic) cluster.

We also computed the average intercluster correlation between the three pairs of clusters, as the simple average of all correlation values between all pairs of nodes that belong to the two clusters. The values are −0.04 between clusters 1 and 2, −0.05 between clusters 1 and 3, and −0.20 between clusters 2 and 3.

**Figure 2:**
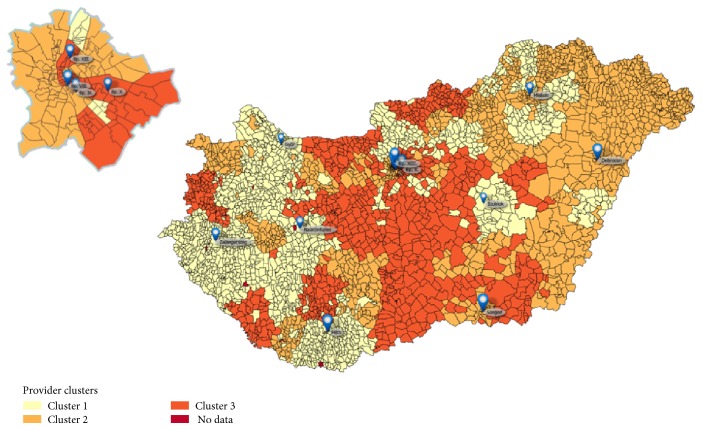
Position of health-care centers belonging to the three clusters. The capital (Budapest), home of about 20% of the total population, is enlarged at the top left. The blue balloon markers show the major tertiary care clinical centers with at least a total of 3000 tertiary cases in the observation period.

We have also observed a correlation between the spatial position of health-care centers and the cluster membership (see Figure 2). Cluster 1 was dominant in Western Hungary, cluster 2 in Eastern Hungary, and cluster 3 in Central Hungary. This fact is quite remarkable because the center characterization method used no geographical information. The map in Figure 2 also shows the major tertiary centers at the blue markers. Some of the tertiary centers are run by a local medical university in the biggest cities of the country like Budapest, the capital, Pécs, Szeged, or Debrecen. For the sake of anonymity, the most important local medical universities will be referred to by the codes of University “A,” “B,” “C,” and “D.” We think that medical universities are important because they can exert a strong influence on the accepted standards of professional conduct at clinics.

Tables 3 and 4 show the numerical characteristics of the clusters. The average cost per patient was computed using the financial data in Table 2.  Table 4 highlights the relative differences among the clusters using the data of Table 3.

The results are evaluated in Discussion. However, the geographical and numerical results of Figures 1 and 2 and Tables 3 and 4 can be summarized as follows.

- Cluster 1 has a relative preference for invasive imaging, proven by the high proportion of “I” and “I-E” event series types. The cluster is dominant in Western Hungary. It includes the clinic of the C university. This cluster has the highest intracluster average edge weight which means strong internal connections, shown also by the strong green patches in the heat map in Figure 1.
- Cluster 2 has a relative preference for noninvasive imaging (“NI” and “NI-E” types), and it is dominant in Eastern Hungary. It includes the clinics of both the A and B universities. This cluster has the lowest intracluster average edge weight, that is, this is the most “diffused” cluster of the three.
- Cluster 3 has a relative preference for invasive treatment followed by noninvasive imaging (“I-NI” type), and it is dominant in Central Hungary. It includes the clinic of the D university. This cluster has high intracluster average edge weight.

Using the financial data in Table 2, we computed the average reimbursement cost of an event series in each cluster. The result for cluster 1 was 75,783 HUF (€ 286), for cluster 2, it was 54,182 HUF (€ 205), and for cluster 3 it was 66,953 HUF (€ 253).

In order to test the robustness of the clustering, we have also processed our network using several other different clustering methods as well, with the following results:

- The Markov cluster algorithm [32], a random walk-based clustering method, gave almost the same result.
- The K-means clustering [33], a vector quantization method, provides 21 clusters as subnetworks of our 3 clusters.
- The ModuLand tool is able to determine hierarchical layers of overlapping network modules [34]. When used this tool on our network, it produced 37 clusters at the hierarchical level 0 which were subnetworks of our clusters, and it produced only 1 cluster with all nodes at the hierarchical level 1.
- We also tried two another modularity-based algorithms: smart local moving algorithm [22] and multilevel local search algorithm [35]; these produced completely the same results.

The next stage in cluster analysis was the test of the nodes' relative importance in the three clusters based on node degrees and node betweenness centralities. We found that the university clinics A, B, C, and D are always located in the top 30% but are never in the top 10% of the strongest members in their cluster. The same holds when we consider the whole network of 136 nodes, so this behavior may be a scale-free feature of university clinics. Budapest, the capital located in the middle of Hungary, has 18 clinics with various clinical pathway spectra. All of three clusters have some health-care centers in Budapest.

Finally, the second level clustering of cluster 2 resulted in two subclusters; the university clinics A and B were placed in the same subcluster which also had a stronger cohesion than the other one. Only at third level clustering were the A and B clinics placed in two different sub-subclusters.

## 4. Discussion

As the results show, we found clear network type relations in the selection of patient evaluation pathways, which was also related strongly to the geographic location of the institutions. It is a reasonable assumption that the decision patterns of individual primary care decision-makers are influenced by the patterns used in their neighborhood. This is why we applied the tools of network analysis. Though the idea is quite straightforward, such methods have not been yet used in the field of health-care pattern analysis, to the best of our knowledge. In the healthcare domain, widely known application fields of network science are gene coexpression network research and microarray studies [36–38]. In these studies, a threshold or cutoff value, usually above 0.6, is normally used for the absolute value of edge weights in the network, below which the edge is not considered present. The aim of using cutoff values is to simplify the network and strengthen the statistical features. In our study, we applied no cutoff values because the three clusters were rather different even without thresholding. This feature shows a strong network organizer effect and lends robustness to our network building algorithm. The robustness of the clustering step was also shown by the cluster assignments being quite independent from the edge weight transform formula. The Louvain method for clustering proved a good choice as it provided a low number of clusters with good characteristics, independently from the nonlinear transforms of the correlation coefficient. Also, the results were confirmed by other clustering methods as well.

We can regard the averaged intercluster Pearson correlations as measure of similarity between two clusters. Though strong negative correlation could mean a strong inverse relation in other domains, in our case, the negative values show even less similarity in the health-care process methodology. The measured, close to zero intercluster values show that there is a weak similarity between clusters 1 and 2 and also between clusters 1 and 3, and the stronger negative correlation of −0.20 shows an even weaker connection between clusters 2 and 3.

According to the financial results, though cluster 1 and cluster 2 have a similar population demography, there is a considerable difference between average care costs as cluster 1 (the “invasive” cluster) has a 28.5% higher average cost per patient than cluster2 (the “noninvasive” cluster). This is not surprising considering the several high-cost invasive events in the event series.

It is also not surprising that each cluster contains at least one major medical university and a tertiary center run by the university. The fact that university clinics are strongly linked to the other cluster members and they are among the 30% most important “opinion leader” nodes in their cluster further supports the assumption that medical universities may have a stronger impact on the distribution of health-care pathways, and therefore on the real clinical practice, than official professional guidelines or protocols.

The correlation between the spatial position of health-care centers and cluster membership suggests that there is a kind of local information spread between neighboring institutions. Another finding that supports this hypothesis is that in all of the cities like Budapest, Debrecen, Szeged, Miskolc, and Pécs, there are at least two clinics with almost the same clinical pathway distribution.

The resulting three clusters can be characterized as follows.

1. Cluster 1 (the “invasive” cluster) has a much higher revascularization rate than cluster 2 (*p* < 0.01), but the 365-day mortality rates for the two clusters are almost the same (*p* < 0.05) according to Table 3. This indicates that in many cases, the revascularization procedure may be unsuccessful or unnecessary. The deficient impact of revascularization procedures on the survival of patients with stable coronary artery disease was demonstrated several years ago by multinational, multicenter randomized studies like [38], but this result had hardly any consequence in the clinical practice. We can be sure that also in our country, a great proportion of patients who underwent coronary angiography and subsequent coronary revascularization had no documented severe myocardial perfusion abnormalities. In such cases, the invasive procedures increase the periprocedural risk of patients without a clear, long-term beneficial effect [39].
2. Cluster 2 is the most diffused cluster, and the only one which includes clinics of two different medical universities (universities A and B). Hierarchical cluster analysis has shown that these two clinics are indeed closely connected. The background of this close relation is clearly connected to history of the B center. The head of this center spends the first two decades of his/her carrier in the A center, while the third and fourth decades in the B center. The other subcluster is centered around a new subsidiary institution of center B working since the middle of the observation period of this study.
3. Cluster 3 is quite different from the other two clusters. The somewhat strange pattern of invasive procedures followed by noninvasive ones is an admixture of the two previous patterns. The physicians in this cluster prefer to start directly with an invasive procedure, but they are very careful in the follow-up of the patients. The mortality rate is significantly higher than in the other clusters. The average age of patients is also significantly higher, which can in part explain both the increased mortality and the biased evaluation pattern.

The strength of the study and the conclusions are that Hungary has a unified, free health insurance system operated by the state; the share of the private sector in our field of interest is negligible; therefore, the input data can be considered complete for the whole population.

There are several limitations of the approach presented. Though the input data that we used spans five years ending in 2008, we considered the health-care system “static” in the analysis, that is, the effect of changes occurring in the system during the period, such as new care centers entering the system, was neglected. At the data preparation phase, the voting scheme that assigns a ZIP area to a single “dominant” care provider may produce distorted results in areas where two or more strong providers compete; however, we felt that sharing ZIP areas among the providers would overcomplicate the analysis. In the analysis, we use mortality ratios, influenced to some extent by the chosen clinical pathway itself, to characterize the providers and clusters. However, as we argued in [4], this influence should be rather limited as revascularization procedures hardly affect survival. Finally, we characterize each center as a single entity though several doctors working in the institution, in spite of being in close day-to-day professional communication, may follow different practices.

## 5. Conclusion

Using a totally data-driven method, we observed in our study that despite national and international clinical guidelines, there are strong regional patterns in medical practice.

The significantly different regional behavior in the care methodology has quantifiable consequences in terms of care costs and periprocedural risk of patients as significantly higher revascularization rates and clinical procedure costs are coupled with almost identical 365-day mortality rates. These results may call for review of the revascularization practices in some parts of the country.

Our network analysis of the care system has also shown that doctors are social people who intensively communicate professional issues. As we observed, medical universities with their university clinics can act as opinion leaders and thus have an important role in shaping the care process.

Further work in the field includes analyzing whether the differences in the available care facilities at the centers have an impact on the costs associated with the clinical care.

## Figures and Tables

**Table 1:** Data summary: patient numbers and 365-day relative mortality of different clinical event series types.

Event series type	% of patients (%) (*n* = 506087)	Relative mortality (%) (*n* = 7543)
E	76.11	0.77
E-NI	3.33	0.76
E-NI-I	0.70	0.64
E-I	4.15	1.62
E-I-NI	0.12	0.45
NI	5.00	2.33
NI-E	0.09	1.19
NI-E-I	0.02	0.72
NI-I	0.88	2.83
NI-I-E	0.11	0.71
I	7.80	8.03
I-E	1.36	1.64
I-E-NI	0.04	1.75
I-NI	0.19	4.56
I-NI-E	0.02	N/A

**Table 2:** Reimbursement costs of events in the event series.

Diagnostic or therapeutic event type	Associated reimbursement cost	
	HUF	Euro
Stress electrocardiography	3408	13
Stress echocardiography	12,962	49
Single photon emission computed tomography	35,379	134
Coronary angiography	145,274	549
Percutaneous coronary intervention	804,834	3040
Coronary artery bypass grafting	1,262,914	4770

**Table 3:** Upper section: distribution of event series types for each cluster and the whole population. The lower section contains outcome parameters: revascularization rate, 365-day mortality rate, and the average cost of treatment of the patients for each cluster and the whole population. Bottom line: average intracluster correlation coefficient for the cluster.

Pathway type	Cluster 1 *n* = 130327	Cluster 2 *n* = 217514	Cluster 3 *n* = 158246	Whole population *n* = 506087
E (%)	75.61	76.49	75.99	76.11
E-NI (%)	2.32	3.84	3.44	3.33
E-NI-I (%)	0.51	0.8	0.71	0.70
E-I (%)	5.09	3.39	4.41	4.15
E-I-NI (%)	0.08	0.12	0.17	0.12
NI (%)	3.27	6.55	4.3	5.00
NI-E (%)	0.02	0.14	0.08	0.09
NI-E-I (%)	0.01	0.04	0.02	0.02
NI-I (%)	0.58	1.15	0.75	0.88
NI-I-E (%)	0.09	0.12	0.11	0.11
I (%)	10.28	6.04	8.18	7.80
I-E (%)	1.88	1.02	1.4	1.36
I-E-NI (%)	0.03	0.04	0.05	0.04
I-NI (%)	0.16	0.17	0.26	0.19
I-NI-E (%)	0.01	0.01	0.05	0.02

REVASC R. (%)	4.63	3.09	4.05	3.79
MORT. (%)	1.38	1.45	1.61	1.48
AVG COST (HUF)	75,783	54,182	66,953	63,738

AVG CORR.	0.38	0.12	0.37	N/A

**Table 4:** Percent rate differences of cluster features compared to each other in pairs. For an explanation on features, see Table 3 caption.

Pathway	Cluster 1 versus cluster 2	Cluster 1 versus cluster 3	Cluster 2 versus cluster 3
E	−1.14% (*p* < 0.05)	−0.5% (*p* = 0.37)	+0.65% (*p* = 0.19)
E-NI	−39.58% (*p* < 0.01)	−32.5% (*p* < 0.01)	+11.7% (*p* < 0.01)
E-NI-I	−36.64% (*p* < 0.01)	−28.7% (*p* < 0.01)	+12.75% (*p* < 0.01)
E-I	+50.17% (*p* < 0.01)	+15.36% (*p* < 0.01)	−23.18% (*p* < 0.01)
E-I-NI	−34.2% (*p* < 0.01)	−51.6% (*p* < 0.01)	−26.44% (*p* < 0.01)
NI	−50.08% (*p* < 0.01)	−23.92% (*p* < 0.01)	+52.43% (*p* < 0.01)
NI-E	−79.93% (*p* < 0.01)	−66.21% (*p* < 0.01)	+68.89% (*p* < 0.01)
NI-E-I	−86.13% (*p* < 0.01)	−70.81% (*p* < 0.01)	+111.6% (*p* < 0.01)
NI-I	−49.48% (*p* < 0.01)	−22.91% (*p* < 0.01)	+52.64% (*p* < 0.01)
NI-I-E	−21.33% (*p* < 0.05)	−14.47% (*p* = 0.2)	+8.71% (*p* = 0.41)
I	+70.15% (*p* < 0.01)	+25.66% (*p* < 0.01)	−26.14% (*p* < 0.01)
I-E	+83.1% (*p* < 0.01)	+33.63% (*p* < 0.01)	−27.01% (*p* < 0.01)
I-E-NI	−21.76% (*p* = 0.19)	−37.27% (*p* < 0.01)	−19.72% (*p* = 0.15)
I-NI	−3.38% (*p* = 0.7)	−37.42% (*p* < 0.01)	−35.23% (*p* < 0.01)
I-NI-E	−37.93% (*p* = 0.31)	−89.69% (*p* < 0.01)	−83.34% (*p* < 0.01)
REVASC R.	+49.39% (*p* < 0.01)	+14.25% (*p* < 0.01)	−23.57% (*p* < 0.01)

MORT.	−5.67% (*p* = 0.18)	−15.25% (*p* < 0.01)	−10.16% (*p* < 0.01)
AVG COST	+39.86%	+13.18%	−19.07%

AVG CORR.	+219.64%	+1.68%	−68.11%

## Abbreviations

AMI: Acute myocardial infarction
CA: Coronary angiography
CABG: Coronary artery bypass grafting
ICD: International classification of diseases
ICPM: International classification of procedures in medicine
IHD: Ischemic heart disease
PCI: Percutaneous coronary intervention
SPECT: Single-photon emission computed tomography.
